# Molecular Farming in *Artemisia annua*, a Promising Approach to Improve Anti-malarial Drug Production

**DOI:** 10.3389/fpls.2016.00329

**Published:** 2016-03-18

**Authors:** Giuseppe Pulice, Soraya Pelaz, Luis Matías-Hernández

**Affiliations:** ^1^Sequentia Biotech, Parc Científic de BarcelonaBarcelona, Spain; ^2^Plant Development and Signal Transduction Department, Centre for Research in Agricultural GenomicsBarcelona, Spain; ^3^Institució Catalana de Recerca i Estudis AvançatsBarcelona, Spain

**Keywords:** biofarming, malaria resistance, *Artemisia annua*, artemisinin, transcription factors, hormones, genetic engineering

## Abstract

Malaria is a parasite infection affecting millions of people worldwide. Even though progress has been made in prevention and treatment of the disease; an estimated 214 million cases of malaria occurred in 2015, resulting in 438,000 estimated deaths; most of them occurring in Africa among children under the age of five. This article aims to review the epidemiology, future risk factors and current treatments of malaria, with particular focus on the promising potential of molecular farming that uses metabolic engineering in plants as an effective anti-malarial solution. Malaria represents an example of how a health problem may, on one hand, influence the proper development of a country, due to its burden of the disease. On the other hand, it constitutes an opportunity for lucrative business of diverse stakeholders. In contrast, plant biofarming is proposed here as a sustainable, promising, alternative for the production, not only of natural herbal repellents for malaria prevention but also for the production of sustainable anti-malarial drugs, like artemisinin (AN), used for primary parasite infection treatments. AN, a sesquiterpene lactone, is a natural anti-malarial compound that can be found in *Artemisia annua*. However, the low concentration of AN in the plant makes this molecule relatively expensive and difficult to produce in order to meet the current worldwide demand of Artemisinin Combination Therapies (ACTs), especially for economically disadvantaged people in developing countries. The biosynthetic pathway of AN, a process that takes place only in glandular secretory trichomes of *A. annua*, is relatively well elucidated. Significant efforts have been made using plant genetic engineering to increase production of this compound. These include diverse genetic manipulation approaches, such as studies on diverse transcription factors which have been shown to regulate the AN genetic pathway and other biological processes. Results look promising; however, further efforts should be addressed toward optimization of the most cost-effective biofarming approaches for synthesis and production of medicines against the malaria parasite.

## Introduction

Malaria is a parasite infection that still affects millions of people worldwide. According to the annual World Health Organization (WHO) report (WHO, 2014b, Malaria World Report), about 90% of all malaria deaths occur in Africa, mostly among children under the age of five. Therefore, malaria has been listed among the most significant causes of death worldwide (WHO, 2014b; Malaria World Report). Malaria is a protozoan disease, transmitted by mosquitoes of the genus *Anopheles.* Among the four species of the *Plasmodium* genus that provoke malarial infections in humans, most cases relate to either *Plasmodium vivax* or *P. falciparum;* the latter being the most common and responsible for almost all of the deaths (White et al., 2014). Despite malaria being eradicated from the USA, Canada, Europe, and Russia, its incidence increased, especially in tropical countries, from the ‘1970s through the ‘1990s of the last century. Since then, new progresses in terms of prevention and treatment have been developed, in an attempt to control and eliminate the infection. However the number of affected people, and deaths, still remain high, and the disease is currently transmitted in 108 countries worldwide (Feachem et al., 2010; Alonso et al., 2011). There are three major reasons for the high persistency of malaria. First, the onset of resistance to anti-malarial drugs: the *Plasmodium* parasite developed resistance to different treatments, especially when only a single drug, quinine, was being administered (White and Olliaro, 1996). This evidence pushed toward the search for new treatments, while WHO (2008) suggested the use of “combination therapies” to treat malaria. The discovery of AN, an anti-malarial compound found in *A. annua*, and its use in the “combination of anti-malarial treatments” (ACT), has guaranteed a very powerful and efficacious ACT. Indeed, the Nobel Prize in Medicine 2015 has been recently awarded to, among others, Professor Youyou Tu for her discovery of this anti-malarial compound. Despite this, two main issues related to ACT still remain unresolved. Production cannot cover the increasing demand in countries where the disease is endemic, while the cost of these drugs is very high for the people who need it the most. Plant molecular farming combines agriculture and metabolic pathway engineering, exploiting the plant’s natural biochemical pathways. In this context plant biofarming would constitute a better cost-effective and valuable system for producing huge amounts of AN, and thereby improving the social-economic conditions of malaria-affected areas.

A second cause of malaria’s persistency is climate change and migration. These phenomena interact with environmental factors, causing an increase in the distribution and impact of malaria in endemic countries and an emergence in non-endemic ones (Lindsay and Thomas, 2001; Reiter et al., 2004; Lindsay et al., 2010; Caminade et al., 2014; Roiz et al., 2014). During the early part of the 21st century, invasive mosquitoes became widely established across Europe and, for example, malaria reappeared in Greece (Danis et al., 2011, 2013). In addition, pyrimethamin-resistant parasites moved from Southeast Asia, spreading resistance alleles across Africa (Roper et al., 2004). Therefore, analyzing the socio-environmental changes and monitoring migration have become critical tools for keeping the malaria alert alive, even in countries where the presence of this, and other, airborne diseases is absent or almost undetectable.

Finally, a relaxation by health authorities in terms of controlling the spread of the disease has been observed in recent years. In areas where infection is unstable or confined to a period of a few months, a burst of infection may occur – due to climatic or social changes and mixed with a lack of prevention and health care. Consequently, this symptomatic disease, which can occur at all ages, could possibly cause epidemics. As a result, untreated and improperly treated malaria cases may lead to excessive malaria mortality and morbidity.

This review will offer a panoramic view of the onset of drug resistance, which constitutes a major threat against malaria treatment and, hence, its eradication. Additionally, attention will be paid to evaluating and elucidating the contribution and impact of different *A. annua* biofarming approaches, for improving drug production and decreasing its price. This evaluation will include not only promising results but also weaknesses and technological gaps, in an attempt to optimise these approaches and improve the living and working conditions of the inhabitants of the affected areas.

## Malaria Life Cycle, Prevention and Treatment

**FIGURE 1:**
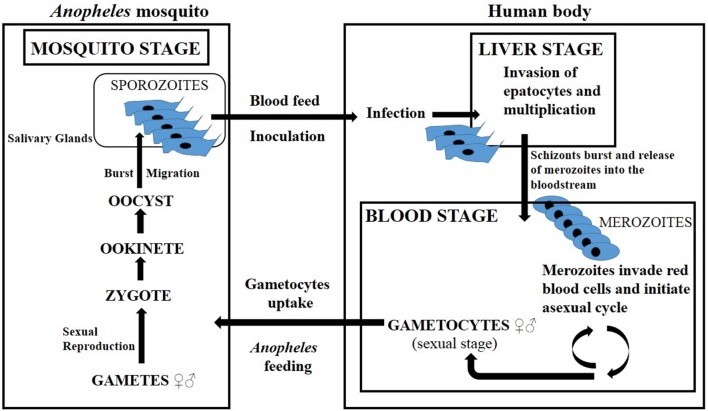
**Lifecycle of *Plasmodium falciparum* (adapted from Cox, 2010).** The cycle starts when a mosquito inoculates motile *Plasmodium* sporozoites, which then move from the dermis to the liver, through the bloodstream. The sporozoites invade the hepatocytes and proliferate. One week after, the liver schizonts burst, releasing a great number of merozoites into the bloodstream. These invade the erythrocytes and start the asexual cycle, with some parasites developing into male and others into female gametocytes (precursors of gametes). After feeding, the *Anopheles* mosquito ingests the gametocytes from the blood. Once inside their vector, they can reproduce sexually, originating in an ookinete and then an oocyst. The oocyst bursts, releasing sporozoites, which migrate to the salivary glands and the cycle is complete.

The stages of the malaria parasites’ life cycle have been recomposed, like a puzzle, through the time, considering that findings have not proceeded in a linear way. This complex history is accurately described in a review by Cox (2010). Updated information about the malarial cycle and disease was taken from White et al. (2014) and is summarized in **Figure 1**. Female *Anopheles* mosquitoes are responsible for the transmission of malaria because they feed on blood (mostly at night), while males feed on plant nectar. A mosquito that is hosting the *Plasmodium* transmits malaria by the inoculation of motile *sporozoites* into the blood of a vertebrate host, and the disease is provoked by the consequences of red-cell parasitisation and destruction. Severe malaria is provoked by massive sequestration and destruction of red blood cells, then finally affects vital organs and causes death. For the purpose of this review, we have summarized all the data in a schematic description (**Figure 1**).

The development of a malaria vaccine has been a very challenging task, especially because of the nature and evolution of the *Plasmodium* infection. In 2015, the European Medicines Agency’s Committee for Medicinal Products for Human Use (EMA-CHMP) expressed, for the first time, a positive scientific judgment in favour of a potential anti-malarial vaccine, the RTS,S/AS01. Its benefits outweigh the risks in the age groups examined and this vaccine may be used in high-transmission areas in which mortality is very high. Unfortunately, the vaccine’s efficacy is limited and it does not offer complete protection (WHO’s Initiative for Vaccine Research; Agnandji et al., 2011; RTS,S and Clinical Trials Partnership, 2012; European Medicines Agency press release, 2015). Therefore, complementary and sustainable strategies are still of paramount importance in reducing the incidence of malaria. These approaches include a combination of physical (mosquito nets) and chemical/biological (repellent oils) measures, as well as increased access to *A. annua* for first-line treatments against the disease. Unfortunately, prevention does not suffice for eradication of malaria and some efficient treatments have also been successfully used to cure severe cases of malaria in recent decades. However, two main issues still exist: the high cost of antimalarial drugs, which the majority of the population in developing countries cannot afford to pay, and the increasing drug-resistance that the *Plasmodium* parasite has developed (Verdrager, 1995; White and Olliaro, 1996). Uncontrolled drug distribution and use, and phenomena such as migratory events and climate changes, have contributed to the development of drug resistance. This is the reason why WHO first recommended the use of a combination of anti-malarial treatments as ACT, based on AN, (WHO, 2008) as an attempt to avoid, or at least reduce, parasite resistance. Unfortunately, improper, widespread use or incorrect prescription management may increase the insurgence of disease resistance (White, 2004; Gbotosho et al., 2009), having a devastating effect on worldwide malaria control. ACT was restricted only to the most difficult cases of malaria but, in 2010, the WHO changed its policy and authorized the use of ACT as first-line treatment at a global level. Major concern was provoked by data indicating that AN resistance had already emerged in small areas of Cambodia, Thailand, Vietnam, and Myanmar (Denis et al., 2006; Noedl et al., 2008; Dondorp et al., 2009; Rogers et al., 2009; Carrara et al., 2013; Leang et al., 2013; Saunders et al., 2014; Tun et al., 2015). This Southern Eastern Asia area may be a possible route for the spread of resistance to the Indian subcontinent (Gething et al., 2011); the same path as that followed by chloroquine in the past (Wellems et al., 2009; Ashley et al., 2014). This evidence raised the level of alarm and the WHO quickly started a campaign in the areas hit by AN resistance, attempting to control the spread and understand how far it had reached. So far, the spread has not affected Africa (Amaratunga et al., 2012; Lopera-Mesa et al., 2013; Ashley et al., 2014; Mok et al., 2015). Fortunately, real-time detection and monitoring of the distribution of drug-resistant malaria parasites can help to prevent the spread (Miotto et al., 2014; WHO, 2014a, Status report on artemisinin resistance, Menard and Ariey, 2015; Tun et al., 2015).

## From Traditional Medicine Toward Biofarming

Malaria inflicts a huge economic burden on individuals and entire communities in developing countries. As a consequence, the high prevalence of malaria in the poorest countries should be a global health priority for the foreseeable future (Sachs and Malaney, 2002; Chima et al., 2003; WHO, 2014b, Malaria World Report).

Malaria prevention is one of the most cost-effective interventions available (White et al., 2011). Indeed, the cost-effectiveness of the different malaria treatments has improved significantly in recent years, using measures for prevention such as the aforementioned mosquito nets and repellents. But this also might be improved upon by the introduction of biofarming and exploitation of plant-based-drugs and different traditional medicines. Indeed, the use of traditional medicine in tandem with modern medicine has been identified as one of the main factors that could explain the significant improvements of health and social indicators in several developing countries (Simpson, 1988; Waxler-Morrison, 1988; Follér, 1989; Montenegro and Stephens, 2006).

**FIGURE 2:**
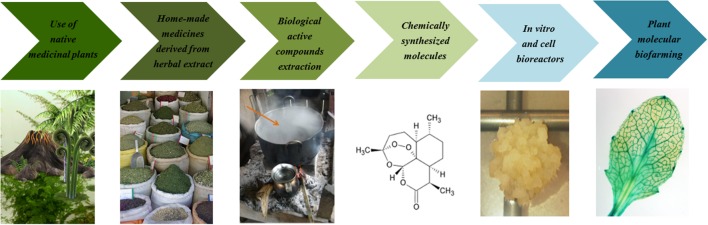
***Pharmacopoeia evolution* across time.** In recent centuries, pharmacopeia has developed from traditional medicine toward into plant biofarming, which integrates the use of traditional medicine with modern medicine; therefore becoming an effective and sustainable approach for improving the well-being and health of a community. Since the use of native medicinal plants and home-made herbal extracts, derived from ethno-botanical local plants, pharmacopeia has moved toward the extraction of useful essential oils and other active compounds with healing properties. Finally, plant molecular pharming has been proposed as a real alternative to chemically synthesized molecules and microbial and mammalian cell bioreactors.

Historically, local pharmacopeia based on native medicinal plants had been adopted by human beings even before society was created (Fernandez, 2006). Since then, pharmacopeia has passed through its own evolution process, starting with ethno-botanical local and home-made medicines, derived from basic herbal extract compounds, followed by chemically synthesized pharmaceutical molecules, and reaching nowadays plant biofarming processes (**Figure 2**). Plant molecular farming combines metabolic pathway engineering and agriculture, in order to use plants as factories and produce valuable products such as recombinant proteins, vaccines or pharmacological molecules (Thomson, 2008; Rybicki, 2014). Indeed, plant biofarming represents an intriguing alternative to microbial and mammalian cell bioreactors. The use of plants instead of microbial and animal cells can significantly reduce the production costs (Webster, 2004). Moreover, most of the molecules produced in plants can be safely stored for long periods without refrigeration, if they are expressed in seeds or leaves that can be stored dried (Ahmad et al., 2012). Therefore, plant biofarming represents an unprecedented opportunity to manufacture affordable modern medicines and make them available at a global scale, particularly in underdeveloped countries where access to medicines and vaccines has historically been limited (Murphy, 2007). Consequently there is an increasing interest in the application of plant biofarming to producing indigenous plant-derived medicines, as well as in the identification of unique medicinal plants and the discovery of new pharmacological active compounds. Traditional plant-based medicines that have been genetically improved for prevention and treatment of malaria represent an appropriate example of biofarming and will be described below.

## Malaria Prevention and Treatment Using Plant-Based Medicines

Hundreds of plants have been identified around the world as potential repellents against diverse types of mosquito. Some of these natural herbal repellents may prevent the bite of *Anopheles*, which is the transmission vector of the malaria parasite (Gupta and Rutledge, 1994). Indeed, research analysis conducted in the past revealed that, among different plants, those that were most used as *Anopheles* mosquito repellents were Neem (*Azadirachta indica*), *Ocinum gratissinum*, *Ocinum suave*, *Eucalyptus camaldulensis*, *Lantana camara*, and *Lippia uckambensis* (Seyoum et al., 2002a,b; Dugassa et al., 2009; Kebede et al., 2010), as reported in **Table 1**.

**Table 1:** Table showing the different medicinal plants used for preventing and treating malaria all along the history.

Most used medicinal plants used to prevent malaria	
Neem (*Azadirachta indica*)	*Eucalyptus camaldulensis*
*Lantana camara*	*Lippia uckambensis*
*Ocinum gratissinum*	*Ocinum suave*
**Medicinal plants used for malaria treatment**	
*Adansonia digitata*	*Ampelozizyphus amazonicus*
*Artemisia annua*	*Aspidosperma rigidum*
*Azadirachta indica*	*Bertholletia excels*
*Ficus sur*	*Cassia alata*
*Cassia occidentalis*	*Cassia siamea*
*Cinchona calisaya*	*Cinchona succirubra*
*Cochlospermum planchonii*	*Plumbago Zeylanica*
*Simaba cedron*	*Tithonia diversifolia*
*Turraea robusta*	*Turraea nilotica*
*Vernonia amigdalina*	

Due to their easy processing, efficiency and reduced cost, these indigenous herbal repellents, if properly used, could become a useful and sustainable approach for reducing malaria-related infections and deaths (Mishra et al., 1995; Ansari and Razdan, 1996; Okumu et al., 2007). Despite this, molecular biofarming has not yet been applied to any of these species. It could be a useful tool for increasing the repellent content of the plant and, thereby, optimize the efficacy of its anti-malarial properties. Consequently, future efforts may focus on the potential of molecular farming for improving the repellent activity of these plants.

Unfortunately, as prevention alone is not enough for the eradication of malaria, treatment has become a crucial approach for prevention of death. Some efficient treatments have been used in recent decades; however, in most of the countries where malaria existence was reported, the malaria parasite developed resistance to quinine (White and Olliaro, 1996). Quinine is a substance isolated from the peruvian trees *Cinchona calisaya* and *Cinchona succirubra* and was used for almost four centuries as the main drug for malaria treatment. Plant-derived products keep making huge contributions toward the fight against malaria, either as known, direct, anti-malarial agents or as potential, and more efficient, novel anti-malarial compounds (Kumar et al., 2009). Indeed, not only the *Cinchona* tree but other plants species have also been found to have pharmacological properties against the malaria parasite (**Table 1**). This is the case of an herbal remedy based on three plants: *Cochlospermum planchonii*, *Phyllanthus amarus*, and *Cassia alata* (Kaushik et al., 2015; Lamien-Meda et al., 2015). *C. planchonii* roots alleviate malarial symptoms, while *P. amarus* and *C. alata* leaves and aerial tissues have anti-malarial activity. Moreover, the active compounds of these three plant species are able to act synergistically as a proper anti-malarial phyto-medicine (Kaushik et al., 2015; Lamien-Meda et al., 2015). Similar results have been found within the diverse range of indigenous Amazonian plants so far studied: *Aspidosperma rigidum*, *Ampelozizyphus amazonicus, Bertholletia excels*, and *Simaba cedron* have all been found to contain the most active anti-malarial extracts among the amazon plants tested to date (Frausin et al., 2015; Oliveira et al., 2015) (**Table 1**). Interestingly, it is not only species in the Asiatic and Amazonian sub-areas, but Sub-Saharan Africa’s enormous plant biodiversity is also proving to be a source of new anti-malarial phyto-remedies. Useful chemical compounds, with anti-plasmodial activity, efficacy and safety have been found in endemic plants growing in this area, including *Adansonia digitata*, *Azadirachta indica*, *Ficus sur*, *Cassia occidentalis*, *Cassia siamea*, *Nauclea latifolia*, *Plumbago Zeylanica*, *Tithonia diversifolia, Turraea robusta*, *Turraea nilotica*, and *Vernonia amygdalina* (Chinsembu, 2015; Irungu et al., 2015). However, excluding quinine, due to the insurgence of resistance to it, and all the potential therapeutic plants already described, nowadays the most widely used and efficient ACTs are those combining natural AN or chemically AN-synthesized derivates, such as artesunate and artemether (Ajayi et al., 2008; WHO, 2014b).

## *Artemisia annua* Plant for Treating Malaria

Artemisinin is an anti-malarial compound that can only be found naturally in *A. annua*. Knowledge of the medicinal properties of this plant dates back to the year 168 B.C., when it was first used as a medicinal tea infusion to treat intermittent fevers (De Ridder et al., 2008). Since then, *A. annua* has been used in traditional Chinese medicine to treat malaria and other diseases (Heide, 2006). Due to its unique mode of action, AN is effective against the asexual stage of the malaria parasite’s life cycle (Fidock, 2010). Interestingly, AN is a potential therapeutic agent not only against this parasitic disease but also against viral diseases, the treatment of certain cancers and the reduction of angiogenesis (Efferth et al., 2002; Singh and Lai, 2004; Romero et al., 2005). AN has proven cytotoxic effects against different types of cancer cells, such as breast, colon, renal, ovarian, prostate, central nervous system, leukemia and melanoma cancer cells (Efferth et al., 2002; Ho et al., 2014; Tang et al., 2015). The drug uses diverse mechanisms, such as inducing cell cycle arrest, promoting apoptosis, triggering cancer invasion and metastasis, and preventing angiogenesis, in order to function (Ho et al., 2014).

Chemically, AN is a sesquiterpene lactone compound, that is produced and stored exclusively in *A. annua* trichomes; which are small, isolated, epidermal protuberances on the surfaces of leaves and represent the aerial organs of most vascular plants (Olsson et al., 2009). Trichomes are involved in defending the plants against insect herbivores, viruses, UV light and/or excessive water loss (Traw and Bergelson, 2003). There are several different kinds of trichomes, but they are mainly classified into non-glandular and glandular (Traw and Bergelson, 2003). In *A. annua*, non-glandular trichomes are involved in water absorption, UV-light reflection and seed dispersal. Glandular trichomes have the morphological peculiarity to synthesize, store, and secrete large amounts of specialized and sometimes toxic secondary metabolites, including AN, that protect the plant from predators without interfering with normal plant growth (Wu et al., 2010; Lange and Ahkami, 2013). Glandular secretory trichomes from *A. annua* are formed from ten cells in five pairs; two basal cells, two stalk cells, four sub-apical cells and two apical cells. AN synthesis takes place in the sub-apical and apical cells, while its accumulation is localized in the sub-cuticular space of the trichomes (Duke et al., 1994; Ferreira et al., 1995; Olsson et al., 2009). *A. annua* is a plant that can be readily grown in many environments. However, the AN content extracted from fresh and/or dry leaves is extremely low (0.1–10 mg/g dry weight), as it is only produced in the trichomes. On the other hand, despite the modernization of different techniques, to chemically synthesize the molecule makes the price too high for a significant number of malarial victims; especially those in developing countries where the malarial burden and impact are the greatest (Abdin et al., 2003; Zeng et al., 2008; WHO, 2014a). In the last ten years, the amount of ACTs produced and provided has increased 36-fold (WHO, 2014b). Unfortunately, production still cannot cover the increasing demand of artemisinin-based therapies in endemic countries. Therefore it is critical to improve the AN yield *in planta*, and develop better methods for its production. In order to realize this aim, biofarming has become an essential tool for increasing the worldwide supply of AN in the last decade.

## *Artemisia annua* Biofarming Approaches Using Metabolic Engineering

**FIGURE 3:**
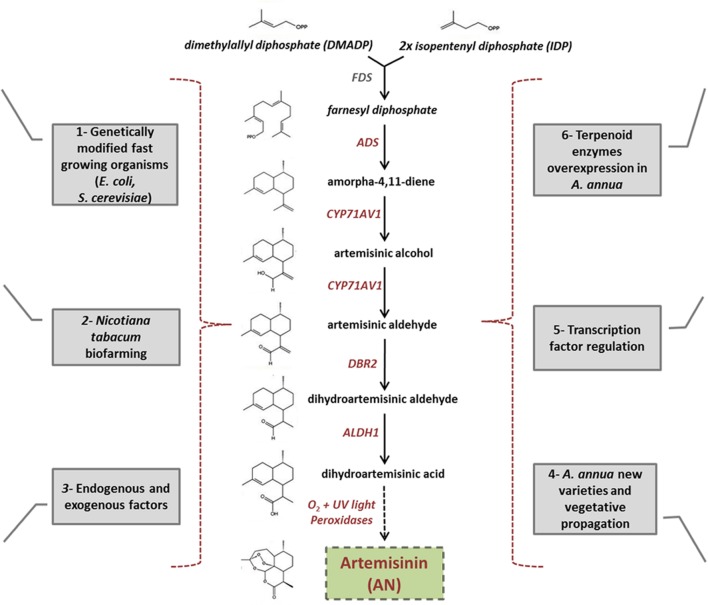
**Diagram showing the different *biofarming* alternatives used instead of AN chemical synthesis. (1)** Use of genetically modified, fast growing organisms, such as *Escherichia coli* and *Saccharomyces cerevisiae*, for AN production. **(2)** *Nicotiana tabacum* used as a model plant for molecular farming. **(3)** Plant endogenous factors, such as phytohormones and external abiotic factors, positively affect both trichome proliferation and artemisinin biosynthesis. **(4)** Crop-breeding and vegetative propagation used to obtain tissue with higher AN content **(5)** Use of transcription factors, that positively regulate artemisinin pathway, as an approach for increasing the amount of this molecule in the plant. **(6)** Metabolic engineering of overexpressing endogenous enzymes of terpenoid biosynthesis in *A. annua.* The artemisinin biosynthetic pathway occurs exclusively in the glandular trichomes of *A. annua* plant. Despite AN biosynthesis utilizes carbon mainly from the mevalonate pathway in the cytosol, Farnesyl diphosphate (FPP), AN precursor, is partially synthetized in the plastid through a non-mevalonate pathway. Therefore, redirection of the mevalonate pathway away from the cytosol, to plastid compartments, such as chloroplasts and mitochondria, may be a novel approach for increasing significantly AN production.

A prerequisite for the success of any secondary metabolite production using metabolic engineering is a deep understanding of its synthesis at the genetic level. For this reason, biochemical and molecular biological studies have been able to elucidate the complete biosynthetic pathway of AN in *A. annua;* as schematically reported in **Figure 3**. Genes encoding components of this pathway are specifically expressed in the *A. annua* trichomes located on leaves, floral buds, and flowers (Olsson et al., 2009). Two molecules of isopentenyl diphosphate (IDP) and one molecule of dimethylallyl diphosphate (DMADP) are condensed by farnesyl diphosphate synthase (FDS) to obtain farnesyl diphosphate (FDP). FDP, which generally serves as a precursor for sesquiterpenes including AN, is then converted into amorpha-4,11-diene through the activity of amorpha-4,11- diene synthase (ADS), and this is the first step of AN biosynthesis proper (Bouwmeester et al., 1999; Mercke et al., 2000). Amorpha-4,11-diene is then oxidized in three steps to artemisinic acid, through the action of amorpha-4,11-diene 12-hydroxylase (CYP71AV1), and a single cytochrome P450 monooxygenase (Ro et al., 2006; Teoh et al., 2006). Recent reports have shown that a double bond reductase (DBR2) and an aldehyde dehydrogenase (ALDH1) operate in the conversion of artemisinic aldehyde to its dihydro form, and then into the direct AN precursor dihydroartemisinic acid, respectively (Zhang et al., 2008; Teoh et al., 2009). The final production step is considered the result of a non-enzymatic, photo-oxidation reaction (Sy and Brown, 2001; Covello, 2008; Brown, 2010). However, recent results have proposed that a peroxidase enzyme or an alternative series of oxidations (occuring exclusively *in planta*) may in fact catalyze the crucial last reaction that converts the precursor into the valuable AN molecule (Bryant et al., 2015).

Over the decades, significant efforts have been made to increase AN production and reduce costs. Some progress, using diverse biofarming approaches, has been made in terms of increasing the production of this compound. This review will analyze the approaches that have been used in great detail; considering which were more or less successful, and identifying their strengths and weaknesses.

### Genetically Modified Fast Growing Organisms

Nowadays, AN derivates produced through chemical synthesis provide the basis for the most efficient ACTs treatments. Despite this, chemical synthesis of AN is not economically feasible because of the complexity and low yield of the process, in addition to the high prices for the people in need. Therefore, the use of genetically modified, fast-growing organisms, such as genetic engineered *Escherichia coli* and *Saccharomyces cerevisiae*, have arisen as a real alternative to chemical synthesis (Lindahl et al., 2006; Zeng et al., 2008). These organisms represent the most widely used heterologous hosts for the expression of enzymes and reconstitution of natural plant product biosynthetic pathways, as has been previously demonstrated for curcumin and piceatannol production (Zhang et al., 2015a).

To further increase cost-effective AN production, metabolic engineering strategies were used, overexpressing AN synthesis enzymes in these microorganisms (**Figure 3**). Cloning and transfer of the ADS enzyme in *Saccharomyces cerevisiae* allowed for the production of the AN precursor amorpha-4,11-diene in yeast (Lindahl et al., 2006). But the most successful strategy using an *S. cerevisiae* bioengineering approach was accomplished by combining different enzymatic steps (Ro et al., 2006). These steps included the cloning of the farnesyl pyrophosphate (FPP) biosynthetic pathway to increase FPP production, which is the immediate precursor before entering the AN pathway proper. This step was followed by reconstitution of the AN enzymes (mainly ADS and CYPP450) in these FPP high-producer yeasts. Even though a large amount of arteminic acid was produced, the final desired product, AN, was not synthesized. Although the AN biosynthetic pathway has been well investigated and great progress has been made in terms of cloning biosynthetic enzymes, the last step of this peculiar synthesis is not yet completely understood. Due to these previous results, it could be suggested that the last step is probably a typical plant non-enzymatic reaction that cannot be inserted into fast-growing organisms. Therefore, in yeast, AN biosynthesis reaches only production of the precursor, artemisinic acid, which afterward needs to be chemically converted into AN (Ro et al., 2006; Paddon et al., 2013). The large amounts of artemisinic acid that have been produced in yeast could be transformed into AN through semi-synthesis and later purification processes, but with a consequent increase in costs.

In addition to yeast manipulation, genetic engineering using *Escherichia coli* was used as an alternative for AN production in microorganisms. Indeed, the introduction of the mevalonate pathway from *S. cerevisiae* into *E. coli* led to efficient production of terpenoid precursors (Martin et al., 2003). Consequently, *E. coli* might be used as a reliable system for the industrial production of plant sesquiterpenes, considering that some results obtained in the last decade have confirmed the potential of this tool. When a few mevalonate enzymes were heterologously expressed together with the ADS enzyme, this confirmed that amorpha-4,11-diene was produced at high levels in *E. coli* (Martin et al., 2003; Tsuruta et al., 2009). However, as was observed with *S. cerevisiae*, no AN was produced at all, most probably due to the fact that the final step in AN biosynthesis is a plant-only, naturally occurring, reaction.

Considering all the results obtained from production of AN, using microbial hosts such as *S. cerevisiae* and *E. coli*, it can be concluded that further optimization is required to achieve the optimal yield for industrial production. Despite production of AN precursors in these organisms being higher than in wild-type *A. annua*, expensive semi-synthesis and purification processes are needed afterward. Consequently, it was worth exploring more biofarming approaches, in order to improve the cost-effectiveness of AN synthesis and thus reduce the current price of ACT (Ro et al., 2006).

### *Nicotiana tabacum* Biofarming

In recent years, special attention has been paid to the fact that the last step of AN synthesis is not yet completely understood. As stated earlier, it was hypothesized that it could be a non-enzymatic photo-oxidation reaction (Sy and Brown, 2001; Covello, 2008; Brown, 2010). However, recent results have proposed that a peroxidase enzyme, or an alternative series of oxidations that occur exclusively *in planta*, may catalyze this last reaction in the process that produces the valuable AN molecule (Bryant et al., 2015). As a consequence of the partial failure using microbial hosts, and considering that this last step is likely to be restricted to plants, a biofarming approach, using *Nicotiana tabacum*, was introduced to overcome this limitation (**Figure 3**).

*Nicotiana tabacum*, or the tobacco plant, is characterized by fast growth and high biomass production. Therefore it has been already established as a model plant for molecular farming (Kumar et al., 2012). Indeed, isoprenoid metabolic engineering has already been accomplished in tobacco (Kanagarajan et al., 2012; Kumar et al., 2012); strongly suggesting that efficient AN production could be achieved in this way. When heterologous co-expression of the AN biosynthetic pathway enzymes was carried out in tobacco, a clear accumulation of dihydroartemisininc alcohol, but not of artemisinic acid -the precursor to AN was detected (Zhang et al., 2011). This result may be explained by the cellular environment of the transformed tobacco plant. This different cellular environment, in contrast to *A. annua*, may favor biochemical reactions toward the reduction of AN intermediates to alcohols instead of oxidation toward acids (Zhang et al., 2011). Due to this unexpected limitation, further studies taking into account: the nature of the target AN (a unique sesquiterpene lactone); the variability of the possible precursors; the presence of different key genes for regulation; and the cellular environment of the plant used for biofarming should be taken into consideration (Majdi et al., 2015). Current approaches in this field have unfortunately obtained AN yields in tobacco that are a thousand times lower than those obtained in *A. annua* (Paddon et al., 2013). Additionally, chemical synthetic conversion of the AN precursors produced in tobacco is still needed for the last reaction. Therefore, further research in this field is essential, in order to achieve proper cost-effectiveness of AN production.

### Endogenous and Exogenous Factors that Induce AN Production

**FIGURE 4:**
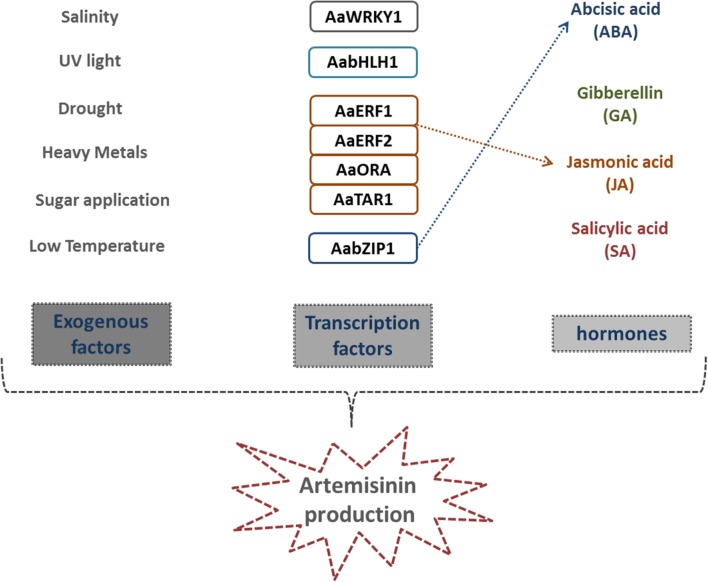
**Essential factors promoting AN biosynthesis.** Endogenous and exogenous factors that positively affect AN biosynthesis. Different abiotic factors inducing plant defense, trichome proliferation and, consequently, AN production. Seven transcription factors have been identified so far in the positive regulation of any of the AN pathway enzymes. Plant hormones ABA, GA, JA, and SA also positively affect both trichome proliferation and artemisinin biosynthesis. Dashed, colored arrows indicate TFs that directly regulate hormone biosynthetic pathways.

Several factors also produced by *A. annua* have been found to positively affect AN synthesis (**Figure 3**). Among these endogenous factors, phytohormones play an essential role. Some plant hormones, such as abscisic acid (ABA), gibberellins (GA), salicylic acid (SA), and jasmonic acid (JA) have been described as positively affecting both trichome proliferation and AN biosynthesis in *A. annua* (Zhang et al., 2005, 2015b; Pu et al., 2009; Yu et al., 2011) (**Figure 4**). JA regulates secondary metabolism in several plant species (van der Fits and Memelink, 2000; De Boer et al., 2011) and, as expected, exogenous treatment with JA also stimulates AN production in *A. annua*, as well as formation of glandular trichomes (Baldi and Dixit, 2008). Similarly, external application of ABA enhances AN production, by stimulating the expression of several synthesis enzymes (Zhang et al., 2009, 2015b). Furthermore, action of SA, a phenolic plant hormone involved in plant development, transpiration, ion uptake and transport, has also been implied in the plant’s response to different abiotic/biotic stresses (Rao and Davis, 1999; Bulgakov et al., 2002; Hayat and Ahmad, 2007). Generally, the mechanisms of plant defense are related to the increase of H_2_O_2_ and reactive oxygen species (ROS) levels (Lamb and Dixon, 1997; Ebel and Mithöfer, 1998). Some studies have revealed that SA applications are able to increase AN content in *A. annua* within a 54% in two different ways: firstly by converting the dihydroartemisinic acid into AN, due to the burst of ROS, and secondly by positively affecting the expression of both AN-related biosynthetic enzymes (Pu et al., 2009).

According to these findings, GA is the hormone that plays the most important role in promoting AN synthesis. In *A. annua*, AN production may in fact increase around 300–400% after exogenous GA treatment, which also positively affects trichome proliferation (Paniego and Giulietti, 1996). Despite the AN and GA pathways taking place in the cytosol and plastids respectively, both of them are well interconnected: an excess of bioactive GA has been described as resulting in carbon being diverted toward an efficient AN production, (Zhang et al., 2005). This assumption is supported by the fact that the levels of transcripts of *FDS*, *ADS* and *CYP71AV1* increase after GA treatment (Banyai et al., 2011; Maes et al., 2011). Consequently, these promising insights regarding exogenous GA application should be taken into consideration as an important tool for future cost-effective AN biosynthesis. Indeed, the GA biosynthetic pathway has been extensively characterized, and most of the genes encoding for the biosynthetic enzymes have been well studied in other plants (Olszewski et al., 2002). Therefore, significant efforts should be addressed toward this complex regulatory network that leads to the final production of the bioactive form of GA. Surprisingly, recent data has shown that GA biosynthetic inhibitors have an interesting, direct, inhibitory effect on *in vitro* growth of the malaria parasite (Toyama et al., 2012). Indeed, treatment with GA inhibitors resulted in morphological changes in the parasite membrane permeability that, if not reversed, fatally injured the parasite (Toyama et al., 2012); revealing an interesting new role for GA in the fight against malaria.

It is also known that AN levels increase with trichome number, density, and maturation. Furthermore, it is known that hormones also control these processes, even if in an independent manner. On one side, ABA, JA, SA and mainly GA control trichome proliferation, by regulating the expression of key *A. annua* Transcription Factors (TFs) that control trichome initiation and AN synthesis - a topic that will be discussed in detail later in this review (Smyth et al., 1990; Dill and Sun, 2001; Kautz et al., 2014; Tian et al., 2014). On the other side, these hormones also interact with and directly control key enzymes of the AN biosynthetic pathway (**Figure 4**).

Not only endogenous hormones but also other substances, such as exogenous sugars, have a positive effect on AN biosynthesis (Arsenault et al., 2010b). However, the role of sugars in AN is complex and sometimes confusing. While sucrose and glucose increase transcript levels of the main AN biosynthesis enzymes, when fructose is added, AN content is significantly reduced (Arsenault et al., 2010b). Additionally, chemical substances such as arsenic, chromium and NaCl also induce AN biosynthesis in *A. annua* (Paul and Shakya, 2013). All these substances were found to significantly increase AN biosynthesis, by affecting the regulation of some of the AN biosynthetic pathway genes. Further evidence strongly suggests that this AN increase is due to the fact that all these substances induce stress in the plant (Paul and Shakya, 2013). Regarding this effect, several reports have shown how exposure of *A. annua* to different abiotic treatments can induce AN biosynthesis. Light, low temperature, salinity, drought, heavy metals and/or other abiotic compounds trigger the generation of active oxygen species (AOS), facilitating the transformation from AN precursors to AN (Wang et al., 2001; Guo et al., 2004; Qureshi et al., 2005; Qian et al., 2007; Pu et al., 2009) (**Figure 4**). The assumption that abiotic stress positively affects AN content in the plant may be explained by the correlation with trichome function. As previously mentioned, the function of both glandular and non-glandular trichomes in *A. annua* is to defend plants against different potential damaging factors using different mechanisms. Consequently, we suggest that all these abiotic factors that are found to increase AN content might be due, at least partially, to an increase in the number of trichomes, as a defense mechanism in response to this stress (Valkama et al., 2004; Wu et al., 2006; Magnan et al., 2008; Sharma et al., 2011). This physiological response may have an indirect effect in the case of glandular trichomes, as this higher trichome density will increase the quantity of secondary metabolites produced. Supporting evidence to this end is that trichomes from many plant species, including *A. annua*, are involved in defending plants against UV light radiation (Traw and Bergelson, 2003), and previous studies have shown how UV light may induce AN production (Rai et al., 2011).

Plants protect themselves from UV stress by producing UV-absorbing compounds, such as flavonoids in the leaf epidermis and trichomes (Rozema et al., 1997; Kumari et al., 2009). Pre-treating *A. annua* plant’s with UV-B and UV-C lead to slight AN increases of 10.5 and 15.7%, respectively. This improvement is not only due to trichome proliferation but also to the alteration of the activity of most of the AN pathway enzymes (Rai et al., 2011). However, considering the low increase in AN production, together with the toxicological UV-C potential, this pre-treatment is not recommended for commercial issues (Rai et al., 2011).

### *Artemisia annua* New Varieties and Vegetative Propagation

Asexual, or vegetative, *in vitro* propagation is a technically easy and cheap method of propagation, used in agriculture and industry for large-scale production of high-value metabolites. It has a several advantages over seed propagation: it retains the genetic constitution of the plant type almost completely and is a less time-consuming process. Moreover, different plant cell cultures can be used for this purpose. So far, *A. annua* cell propagation has been realized using different cell types. *In vitro* propagation of *A. annua* hairy roots has given the best results (Jaziri et al., 1995; Liu et al., 1997) (**Figure 3**). Hairy roots are genetically stable and generally show better biosynthetic potential for secondary metabolites compared with other tissues (Majdi et al., 2015). Interestingly, root hair morphology shares similar genetic regulation to that of trichomes, which may somehow explain these positive results. Furthermore, *in vitro* production of AN can be enhanced by treating cell cultures with different elicitors, such as 2,6-di-*O*-methyl-cyclodextrin (DIMEB) or the aforementioned phytohormones JA, GA and SA (Paniego and Giulietti, 1996; Durante et al., 2011; Majdi et al., 2015). Unfortunately, despite the potential of these tools and the significant effort that has been made regarding *A. annua* cell cultures, none of these methods are commercially available.

During the last decades, *A. Annua* crop-breeding has produced new varieties (**Figure 3**). Indeed, *A. Annua* varieties can be sorted into two chemotypes: the high artemisinin producers (HAPs) and the low artemisinin producers (LAPs) (Brown and Sy, 2004, 2006). HAPs include such varieties as *Chongqing*, *Anamed, Artemis* and *2/39* that produce more AN than arteannuin B, another derivate from artemisinin acid but without therapeutic value (Covello, 2008; Brown, 2010; Reale et al., 2011). These varieties have an average AN content that is twice that of wild type *A. annua* plants. Moreover, the University of York’s Centre for Novel Agricultural Products (CNAP), has recently registered a new HAP variety, Hyb8001R, which will be commercialized in China. Contrarily, LAP varieties produce more arteannuin B than AN and include different *Iran* and *Meise* varieties. Some recent evidence supports the hypothesis that the chemotype is determined mainly by the activity of the DBR2 enzyme activity, whereas HAP varieties show a much higher activity of that enzyme than LAP varieties do (Yang et al., 2015). In order to reach more economically feasible AN production, further studies may focus on the creation of new *A. annua* varieties with an AN content that is much higher than the present varieties.

### Unraveling the *Artemisia annua* Transcription Factor Genetic Engineering Network

Significant but insufficient advances have been made in terms of metabolic engineering for cloning the AN pathway in tobacco, yeast and bacteria (**Figure 3**). However, only a few studies so far have been carried out to study the regulation of this pathway by TFs-encoding genes. Since the first reports of successful *Agrobacterium*-mediated transformation of *A. annua* in the 1990s (Vergauwe et al., 1996; Banerjee et al., 1997), these transformation protocols have been optimized (Han et al., 2005). Generally, TFs regulate the expression of a certain number of genes from specific and/or related pathways (Borevitz et al., 2000); therefore loss and gain of the function of TFs has arisen as a promising biofarming approach for more efficiently regulation of secondary metabolite production (Verpoorte and Memelink, 2002; Petersen, 2007) (**Figure 3**).

In recent years, a few TFs have been characterized as regulators of the transcriptome, for controlling and regulating different enzymatic steps through AN biosynthesis (**Figure 4**). AaWRKY1 was the first TF to be identified and characterized in *A. annua* (Ma et al., 2009). The constitutive and trichome-specific expression of AaWRKY1, driven by the promoters *CaMV35S* and *CYP71AV1*, respectively, dramatically increases the transcript levels of *CYP71AV1*, but does not clearly affect the transcription levels of *FDS*, *ADS*, and *DBR2* (Ma et al., 2009). Additionally, the AaWRKY1 protein can bind the regulatory region of the *ADS* promoter (Han et al., 2014). As a result, in these transgenic plants, AN content is 1.8 times increased compared with wild-type *A. annua* (Ma et al., 2009).

Other studies have revealed how other TFs, belonging to the bHLH and AP2/ERF families; regulate biosynthetic genes (Yu et al., 2011; Lu et al., 2013a; Ji et al., 2014; Tan et al., 2015). *AabHLH1* was isolated from a cDNA library obtained from glandular trichomes in *A. Annua*. Transient overexpression of AabHLH1 in leaves increases expression levels of *ADS* and *CYP71AV1*, the two key enzymes, thereby positively regulating AN biosynthesis. Indeed, biochemical analyses have shown that AabHLH1 protein is able to bind *in vivo* with the E-box *cis*-elements, present in both *ADS* and *CYP71AV1* promoters (Ji et al., 2014). Among all the TFs families, the best characterized in *A. annua* is the AP2/ERF family. Four members of this family *Ethylene Response Factor1* (*AaERF1*), *Aa-Ethylene Response Factor2* (*AaERF2*), *Trichome and Artemisinin Regulator1* (*AaTAR1*) and *AaORA* were found to also directly affect the AN biosynthesis pathway (Yu et al., 2011; Lu et al., 2013a; Tan et al., 2015). Two JA- and ethylene- responsive AP2 family members, AaERF1 and AaERF2, that are highly expressed in *A. annua* inflorescences, are also able to increase twofold the accumulation of AN and artemisinic acid when overexpressed (Yu et al., 2011). In contrast, RNAi lines that partially silence AaERF1 and AaERF2 decrease both content of both metabolites, by directly controlling ADS, CYP71AV1 and moderately DBR2 transcript levels (Yu et al., 2011).

AaTAR1 plays an essential role, not only in regulating AN biosynthesis but also in other biological processes. Similarly to the other TFs described so far, AaTAR1 controls *ADS* and *CYP71AV1* expression, by binding to their regulatory regions. When *AaTAR1* is silenced, AN content is dramatically reduced and cuticular wax distribution is altered (Tan et al., 2015). In addition, AaTAR1 controls both glandular and non-glandular trichome initiation and development in *A. annua*. The last AP2/ERF member studied so far, AaORA, positively regulates the transcript levels of *ADS*, *CYP71AV1*, and *DBR2*, as well as *AaERF1. A*s a consequence, AN content in these plant lines is regulated as well (Lu et al., 2013a). Interestingly, overexpression of AaORA increases the expression levels of diverse genes involved in different, but still related, physiological aspects - such as defense. Phenotypical analyses demonstrate that the AaORA protein is a positive regulator of resistance to *Botryris cinerea*, by modulating the expression of defense marker genes such as PLANT DEFENSIN1.2 (PDF1.2), HEVEIN-LIKE PROTEIN (HEL) and BASIC CHITINASE (B-CHI) (Lu et al., 2013a). Simultaneously, it was found that AaERF1 also confers resistance to *Botrytis cinerea* by activating some of the defense genes via the JA and ethylene signaling pathways in *A. annua* (Lu et al., 2013b).

Recent results obtained using *A. annua* indicate that the TFs identified so far, that function in AN regulation, may be involved in regulating other biological and developmental processes such as trichome proliferation. Since TFs from other plant species are known to be specialized in the regulation of protective secondary metabolite biosynthesis, they are also able to affect phyto-hormone biosynthesis and signaling (Wolucka et al., 2005; Tuteja, 2007; De Boer et al., 2011). There is clear correlation between AN biosynthesis and ABA signaling, as well as overexpression of *AaPYL9;* an ABA receptor ortholog, increases AN production (Zhang et al., 2015b). Moreover, it has been shown recently how AabZIP1, a basic leucine zipper TF, connects ABA signaling to AN accumulation (Zhang et al., 2015b). As previously mentioned, JA-responsive AP2 family members, AaERF1 and AaERF2, directly control expression levels of *ADS*, *CYP71AV1* and, to some extent, *DBR2* (Yu et al., 2011).

Despite significant advances being made into the elucidation of the complex genetic network controlling AN biosynthesis, none of the strategies using loss and gain of TF function has resulted in an efficient enough increase in AN content to be considered for commercial purposes. This limitation might be biochemically explained due to several facts, such as transgenic gene silencing, enzymatic limiting-steps and synthesis/degradation of other final products of the pathway that compete with AN for precursor sources, for example arteannuin B or other terpenoids (Bertea et al., 2005; Zhang et al., 2008; Teoh et al., 2009). Therefore, further studies in this field are essential for dealing with these issues and overcome these difficulties.

### Terpenoid Enzymes Studies in *Artemisia annua*

Due to these limitations, new studies have been conducted to better elucidated the complex regulation of the AN biochemical pathway. For decades, it was believed that FPP precursors (DMAPP and two units of IPP) were only biosynthesized exclusively in the cytosol via mevalonate from acetyl-CoA (Covello, 2008; Arsenault et al., 2010a,b; Graham et al., 2010; Kiran et al., 2010). However, recent results have revealed that despite AN biosynthesis utilizes carbon mainly from the mevalonate pathway, some of the carbon sources for AN synthesis are also provided by plastids (Towler and Weathers, 2007; Schramek et al., 2010). Indeed, a non-mevalonate biosynthetic pathway that occurs in plastids was also found to be a source of terpenes in other plant species, via 1-deoxyxylulose 5-phosphate and 2C-methylerythritol 4-phosphate precursors (Rodriguez-Concepcion, 2006; Rohmer, 2007). In addition, recent studies have provided evidences for metabolic crosstalks between both compartments (Dudareva et al., 2005; Skorupinska-Tudek et al., 2008; Schramek et al., 2010). *A. annua* plants treated with specific inhibitors of mevalonate and non-mevalonate pathway revealed that AN biosynthesis decreased when used any of these inhibitors (Towler and Weathers, 2007); concluding that both pathways are involved in AN formation. Moreover, further studies suggested that DMAPP from cytosolic mevalonate origin is transferred to the plastids, where one of the IPP unit of non-mevalonate origin is used to form geranyl diphosphate (GPP) (Schramek et al., 2010). Once GPP is synthetized, it is exported again to the cytosol other IPP unit but from mevalonate origin it is used to convert GPP to FPP (Schramek et al., 2010).

Consequently, it has been hypothesized that, if carbon resource comes from these two mevalonate and non-mevalonate pathways, some key enzymes from this may also play essential roles in regulating the synthesis and degradation of the final products from the AN pathway. It is well known that for a specific pathway that competes with another one for common precursors, as in this case is the FDP availability, the accelerated conversion of this precursor to the product of interest, using genetic engineering, may minimize the conversion of the precursor to other competing molecules (Majdi et al., 2015). During the past years, a few rate-limiting endogenous enzymes involved in terpenoid biosynthesis have been overexpressed (**Figure 3**). This was the case with *FPS* or isopentyl transferase (IPT) enzymes from *A. annua* and FDP synthase from *Gossypium hirsutum* that were cloned and overexpressed in *A. annua* (Chen et al., 2000). However, and similar to considering the loss and gain of function of TFs, when these enzymes were constitutively overexpressed in the plant, the highest AN content in these transgenic plants was increased two- to three-fold (Ma et al., 2009; Liu et al., 2011; Han et al., 2014). These results indicate, once again, that AN biosynthesis might be strongly regulated by other unknown factors. Indeed, similar evidence was obtained when the expression of squalene synthase and β-caryophyllene synthase – enzymes that compete for FDP with ADS was repressed: *A. annua* plants in which these enzymes were silenced showed a 1.5- to 2-fold increase in AN content (Wang et al., 2012).

Fortunately, when the cellular mevalonate pool and its channelization toward AN biosynthesis was enhanced by overexpression of the enzyme 3-hydroxy-3-methylglutaryl coenzyme A reductase (HMGR), a much better result was obtained (Alam and Abdin, 2011). HMGR is considered to be the rate-limiting enzyme of the mevalonate pathway, that converts HMG-CoA to mevalonic acid at the beginning of isoprenoid biosynthesis in the cytosol (Chappell, 1995; Argolo et al., 2000). Mevalonic acid serves as the common precursor for the synthesis of different secondary metabolites, including: sesquiterpenoids, triterpenoids, sterolsand phytoalexins, from different plant species (Chappell, 1995; Argolo et al., 2000; Ayora-Talavera et al., 2002). When HMGR from *Catharanthus roseaus* was overexpressed, together with ADS, in transgenic *A. annua* plants, AN content increased 7.65-fold (Alam and Abdin, 2011). This result strongly suggests that it is crucial to take into consideration other limiting-rate factors, upstream in the pathway, in order to divert as much carbon resources as possible toward AN biosynthesis. Indeed, the latter strategy was also used in tobacco and showed promising results. When different enzymes, including not only HMGR but also those involved in AN synthesis, were overexpressed in tobacco, AN was finally produced for the first time in another plant (Farhi et al., 2011). Interestingly, in these transgenic tobacco plants, ADS was not only expressed in the cytosol but also in the mitochondria, using a COX4 transit peptide; suggesting the potential of plastid transformation.

## Biofarming *Artemisia annua* Future Strategies

There is great concern among the international health community regarding the onset of AN resistance in the malarial parasite. Even though synthesized AN has been in use for less than 20 years, the first cases of parasite resistance have been already identified. However, *A. annua* tea has been used in china for the last 1000 years without any resistance development. This could be explained by the fact that artificial AN has sometimes been wrongly used as a monotherapy, while *A. annua*, in addition to AN, contains other anti-malarial substances, such as artemetin, casticin, cirsilineol, chrysoplenetin, sesquiterpenes, and flavonoids. These compounds work in synergy with AN, reducing the possibility of the parasite developing resistance (Willcox, 2004). Moreover, it has also been proven that an *A. annua* infusion has the additional effect of strengthening the immune system, which could bring extra benefits to local people in areas affected by malaria (Willcox et al., 2005). The synergic action of the different compounds in *A. annua* suggests that special attention should be directed toward plant biofarming in the future.

Some of the brightest prospects for the success of the plant biofarming field include the plant-made viral vaccines, or desired peptides, that are the earliest products of this new technology (Viana et al., 2012; Rybicki, 2014). The success of these approaches is not only based on the production rate increase of the desired molecule but also on the reproduction of desirable post-translational modifications that reduce the risk of allergenicity (Viana et al., 2012). Unfortunately, early molecular biofarming approaches, based on genetic and metabolic engineering, to increase AN content in different plants and microorganisms have not been as effective as was expected. However, recent reports have highlighted new, promising insights for finally reaching a more cost-effective approach. Genetic and metabolic engineering studies indicate that, despite AN biosynthesis being strictly regulated, it is still possible to modulate it using external factors as well as genetic ones. External application of diverse plant phytohormones, specially GA, or sugar concentrations, together with abiotic factors, could optimize AN production. However, recent results concerning AN production using genetic engineered plants evidence the enormous potential of biofarming for obtaining economically feasible AN synthesis. At the gene level, and similar to biosynthesis of other plant secondary metabolites (Zhang et al., 2015a), overexpressing only the full AN pathway was not sufficient for a significant increase in AN production, and, therefore, rendered AN production in plants uneconomical. This limitation in AN production might be explained by some enzymatic limiting-steps, as well as by competition for precursor sources with other terpenoids, or AN-derivatives, such as arteannuin B. Further studies that have been conducted addressing these issues have revealed promising results. Indeed, evidence has recently shown that the mevalonate pathway could be one of the most efficient biopharming approach used so far to increase AN production using genetic engineering. As strong competition exists among the different pathways for the available mevalonate products, it is therefore crucial to take the rate-limiting factors of carbon diversion, as Alam and Abdin (2011) have proved, into consideration. By overexpressing HMGR and ADS, the rate-limiting enzymes of the mevalonate and AN pathways respectively, *A. annua* plants increase AN content more seven-fold; something that has not been achieved using any other strategies. This evidence suggests that, nowadays, *A. annua* biofarming is finally starting to optimize strategies for production of effective bioengineering AN. Therefore, further research should be addressed toward key-limiting enzymes from other terpenoids precursor sources from either mevalonate or non-mevalonate pathways.

There is also a growing interest in applying proteomics and genomics to *A. Annua*, but one of the biggest handicaps of these techniques is the lack of availability of well-annotated databases. The *in silico* comparison of different *A. annua* databases, including: the EST trichome library; *A. annua* trichome Trinity contig database; Uni/Prot/*A. annua;* and UniProt/*viridiplantae*, have been useful tools for identifying important enzymes and TFs. However, these tools have also revealed significant differences in their suitability for genomic and proteomic analyses. Despite these differences, the EST trichome library has allowed identification of essential proteins, enriched in the *A. annua* trichomes, that are involved in biosynthesis and regulation of AN, as well as other related enzymatic processes (Bryant et al., 2015). Fortunately, the imminent release of the entire genome of *A. annua* will resolve this challenge, provide benefits to the scientific community and offer a better understanding of the genetic machinery regulation for AN production.

Finally, further biofarming efforts should be addressed toward different physiological aspects, such as different plant cell systems and compartments that might be used for large scale production of AN or other useful metabolites. As with many other metabolites of high pharmaceutical value, AN is toxic to the plant itself, as it is able to inhibit cell division and tissue growth (Dayan, 1998). AN is therefore exclusively produced in the glandular trichomes of *A. annua*, since these are independent compartments that are isolated from the rest of the plant. Specifically, the expression of the AN enzymes is active in both apical and subapical cells of the trichomes, while AN and its precursors accumulate in the subcuticular cavity of the glandular trichomes. Future biofarming strategies should also, therefore, pay particular attention to the initiation and development of trichomes. Genetic engineering might be also used as a useful and sustainable tool for AN production, by modifying diverse physiological aspects of the trichomes, such as leaf area, trichome number, density and alteration of the morphology of different cell types that form glandular trichomes. In conclusion, it is essential to keep in mind the use of different subcellular compartments, such as plastids, that could be used as efficient tools to exponentially elevate AN production in different plant species. Studies of different plant species have further revealed that redirection of the mevalonate pathway away from the cytosol, to plastid compartments, such as chloroplasts and mitochondria, is a new and potent approach for increasing sesquiterpene production from 100–10,000 times (van Herpen et al., 2010; Liu et al., 2011). The expression of foreign genes in the chloroplast also allows there to be almost 10,000 genome copies per cell, without the need for a signal peptide or no-gene-silencing possibility, (Bendich, 1987; Hasunuma et al., 2008; Kumar et al., 2012). Consequently, this novel biofarming approach, if properly used, may have enormous potential for economically feasible and sustainable AN production.

## Author Contributions

GP and LM-H conceived and designed the research for this review. GP, SP, and LM-H wrote the manuscript. LM-H. supervised the research and the writing of the manuscript.

## Conflict of Interest Statement

The authors declare that the research was conducted in the absence of any commercial or financial relationships that could be construed as a potential conflict of interest.
